# Kinetics of humoral immune response over 17 months of COVID-19 pandemic in a large cohort of healthcare workers in Spain: the ProHEpiC-19 study

**DOI:** 10.1186/s12879-022-07696-6

**Published:** 2022-09-03

**Authors:** Concepción Violán, Pere Torán-Monserrat, Bibiana Quirant, Noemi Lamonja-Vicente, Lucía A. Carrasco-Ribelles, Carla Chacón, Josep Maria Manresa-Dominguez, Francesc Ramos-Roure, Rosalia Dacosta-Aguayo, Cristina Palacios-Fernández, Albert Roso-Llorach, Aleix Pujol, Dan Ouchi, Mónica Monteagudo, Pilar Montero-Alia, Rosa Garcia-Sierra, Fernando Arméstar, Maria Doladé, Nuria Prat, Josep Maria Bonet, Bonaventura Clotet, Ignacio Blanco, Marc Boigues-Pons, Nemesio Moreno-Millán, Julia G. Prado, Eva María Martínez Cáceres, for the ProHEpiC-19 study, Marta Soldevilla Garcia, Ester Moral Roldan, Magda Alemany Costa, Eva Olivares Ortega, Alba Pachón Camacho, Marta Bujalance Devesa, Mariella Soto Espinoza, Antonio Negrete Palma, Mariana Martinez de San José, Ester Lucas Varas, Ester Badia Perich, Mónica Piña Rodriguez, Elena Domenech Graells, Eduard Moreno Gabriel, Victòria Sabaté Cintas, Mª Jose Argerich González, Asumció Vazquez Duran, Alex Ortega Roca, Anna Devesa Pradells, Athina Kielpilanen, Oscar Blanch Lombarte, Miguel Angel Marin Lopez, Julieta Carabelli, Ruth Peña Poderós, Esther Jimenez Moyano, Eulalia Grau Segura, Laia Bernard Rosa, Raul Pérez Caballero, Felipe Rodriguez Lozano, Gema Fernández Rivas, Sonia Molinos Abos, Jaume Barallat Martinez de Osaba, Lorena Tello Trigo, Cristina Perez Cano, Juan Matllo Aguilar, Anabel López Martínez, Inmaculada Agüera Iglesias

**Affiliations:** 1 Institut Universitari d’Investigació en Atenció Primària Jordi Gol (IDIAP Jordi Gol), Unitat de Suport a la Recerca Metropolitana Nord, Mare de Déu de Guadalupe 2, Planta 1ª, Mataro, 08303 Barcelona, Spain; 2 Direcció d’Atenció Primària Metropolitana Nord Institut Català de Salut, Barcelona, Spain; 3 Germans Trias i Pujol Research Institute (IGTP), Camí de les Escoles, S/N, Badalona, 08916 Barcelona, Spain; 4 Universitat Autònoma de Barcelona, Cerdanyola del Vallès, Spain; 5 Department of Medicine, Faculty of Medicine, Universitat de Girona, 17003 Girona, Spain; 6 Multidisciplinary Research Group in Health and Society GREMSAS (2017 SGR 917), 08007 Barcelona, Spain; 7 Cell Biology, Physiology, Immunology Department, FOCIS Center of Excellence-Universitat Autònoma de Barcelona, Cerdanyola del Vallès, Spain; 8 Immunology Division, Laboratori Clinic Metropolitana Nord (LCMN), Hospital Universitari Germans Trias i Pujol, Badalona, Spain; 9 Fundació Institut Universitari d’Investigació en Atenció Primària Jordi Gol (IDIAP Jordi Gol), Barcelona, Spain; 10 Department of Nursing, Universitat Autònoma de Barcelona, Cerdanyola del Vallès, Spain; 11 Department of Medicine, Faculty of Medicine, Universitat Autónoma de Barcelona, 08193 Bellaterra, Spain; 12 Departament de Pediatria, d’Obstetrícia i Ginecologia i de Medicina Preventiva, Universitat Autónoma de Barcelona, 08193 Bellaterra, Spain; 13 AIDS Research Institute Irsicaixa, Badalona, Spain; 14 Centre d’Atenció Primària La Riera (Mataró 1), Institut Català de la Salut, Barcelona, Spain; 15 Intensive Care Unit, Hospital Universitari Germans Trias i Pujol, Badalona, Spain; 16 Clinical and Biochemical Analysis Division, Laboratori Clinic Metropolitana Nord (LCMN), Hospital Universitari Germans Trias i Pujol, Badalona, Spain; 17 Lluita contra la SIDA Foundation, Hospital Universitari Germans Trias i Pujol, Badalona, Spain; 18 University of Vic-Central University of Catalonia (UVic-UCC), 08500 Vic, Spain; 19 Hospital Universitari Germans Trias i Pujol, Badalona, Spain; 20 Gerència Territorial Metropolitana Nord, Institut Català de la Salut, Barcelona, Spain; 21 Centro de Investigación Biomédica en Red de Enfermedades Infecciosas (CIBERINF), Instituto de Salud Carlos III (ISCIII), Madrid, Spain; 22 Department of Medicine, Faculty of Medicine, Universitat Autònoma de Barcelona, 08193 Bellaterra, Spain

**Keywords:** SARS-CoV-2, COVID-19, Antibodies, IgG, IgM, Seroprevalence, Kinetics, Humoral immunity, Clinical spectrum, Health care workers, Cohort, Non-linear mixed models

## Abstract

**Background:**

Understanding the immune response to the SARS-CoV-2 virus is critical for efficient monitoring and control strategies. The ProHEpic-19 cohort provides a fine-grained description of the kinetics of antibodies after SARS-CoV-2 infection with an exceptional resolution over 17 months.

**Methods:**

We established a cohort of 769 healthcare workers including healthy and infected with SARS-CoV-2 in northern Barcelona to determine the kinetics of the IgM against the nucleocapsid (N) and the IgG against the N and spike (S) of SARS-CoV-2 in infected healthcare workers. The study period was from 5 May 2020 to 11 November 2021.We used non-linear mixed models to investigate the kinetics of IgG and IgM measured at nine time points over 17 months from the date of diagnosis. The model included factors of time, gender, and disease severity (asymptomatic, mild-moderate, severe-critical) to assess their effects and their interactions.

**Findings:**

474 of the 769 participants (61.6%) became infected with SARS-CoV-2. Significant effects of gender and disease severity were found for the levels of all three antibodies. Median IgM(N) levels were already below the positivity threshold in patients with asymptomatic and mild-moderate disease at day 270 after the diagnosis, while IgG(N and S) levels remained positive at least until days 450 and 270, respectively. Kinetic modelling showed a general rise in both IgM(N) and IgG(N) levels up to day 30, followed by a decay with a rate depending on disease severity. IgG(S) levels remained relatively constant from day 15 over time.

**Interpretation:**

IgM(N) and IgG(N, S) SARS-CoV-2 antibodies showed a heterogeneous kinetics over the 17 months. Only the IgG(S) showed a stable increase, and the levels and the kinetics of antibodies varied according to disease severity. The kinetics of IgM and IgG observed over a year also varied by clinical spectrum can be very useful for public health policies around vaccination criteria in adult population.

**Funding:**

Regional Ministry of Health of the Generalitat de Catalunya (Call COVID19-PoC SLT16_04; NCT04885478).

**Supplementary Information:**

The online version contains supplementary material available at 10.1186/s12879-022-07696-6.

## Background

Infection with the SARS-CoV-2 can be detected by measuring the level of virus-specific antibodies, reflecting an immune response against a recent or previous infection [1]. Several studies described the rapid increase (Igs) of various immunoglobulin isotypes (IgA, IgM, IgG) against the epitopes of the spike (S) glycoprotein as well as to the nucleocapsid (N) protein in SARS-CoV-2 infection [2–5].

COVID-19, the disease caused by the SARS-CoV-2, has a broad clinical spectrum, including various forms of clinical presentation ranging between asymptomatic infections and critical illness. Likewise, the antibody response to the SARS-CoV-2 infection is also heterogeneous [6]. Few longitudinal studies have performed serological follow-up across the clinical spectrum, and with a limited study duration between 80 to 270 days [2–4]. An early study reported a rapid rise and subsequent fall of antibodies, which stabilized at later time points, indicating that immunity against SARS-CoV-2 may last for at least 120 days after infection [7]. Two later studies suggested this protection for up to at least 180 days [2, 8] while other recent reports claim that it persists for at least a year [9, 10]. However, knowledge on the kinetics and the immune response to SARS-CoV-2 infection is still limited, and larger and more detailed longitudinal studies are needed to define the half-life of antibodies against SARS-CoV-2.

Of the few studies investigating the kinetics of the antibodies only one have studied IgM antibody [10]. None of these studies have more than five measurements [11]. In addition, all have made either linear or LOESS regression models, which do not allow to obtain a model that represents the non-linearity and heterogeneity of the antibody response, indicated by previous kinetics studies. The use of non-linear models over linear or LOESS models would allow to obtain a more realistic representation of the antibody kinetics that can better explain the behaviour of the antibodies.

The WHO recommends that a generic population-based serological testing should be carried out using enzyme linked immunosorbent assays (ELISA) or immunofluorescence assays (IFA) with standardized reagents [12]. Using reagents in compliance with WHO’s international standards for anti-SARS-CoV-2 antibodies allows to evaluate vaccine efficacy and federate and compare epidemiological and immunological surveillance studies. At the beginning of the pandemic, antibody levels were determined using qualitative assays; while using internationally standardized enzyme immunoassays for the quantitative detection of specific IgG antibodies is more recent [13]. Studies with more sample points and more accurate statistics analysis provide a better assessment of the disease burden and transmission to better inform public health efforts against COVID-19 [9, 14].

The aim of this study is to describe the kinetics of IgM (N) and IgG (N, S) antibodies against SARS-CoV-2 and assess their relationship with the clinical spectrum and other factors. Our findings provide a detailed picture of the immune response against the virus in a population which plays the most crucial role in fighting for the pandemic. Our results can guide public health policies to develop more efficient strategies for monitoring, treatment, and prevention of COVID-19.

## Methods

### Study design and ethics

*ProHEpiC-19* is a prospective, longitudinal study, involving two groups of healthcare workers (healthy and infected) in the Northern Metropolitan Area of Barcelona (Spain). The ethics committees of the Foundation University Institute for Primary Health Care Research Jordi Gol i Gurina (IDIAPJGol) (ref. 20/067) and The Germans Trias i Pujol Research Institute (IGTP) (ref.COV20/00660 (PI-20-205)) approved the study protocol, which has been published on ClinicalTrials.gov (NCT04885478, registered on 13/5/21). All participants recruited in the study were fully informed about the ProHEpiC-19 protocol and signed informed consent to participate. They consented to use their collected data for research and agreed to the applicable regulations, privacy policies, and terms of use. Participant data has been anonymised according to a numerical coding system based on order and stored in a database securely. The database will be maintained for a period of 15 years after the completion of the study.

No participants or members of the public were directly involved in the design or analysis of the reported data. The funders of the study had no role in study design, data collection, data analysis, data interpretation, or writing of the report. The corresponding author (CV) had full access to all data, while LACR, ARLL, DO, JMMD, and AP; had access to the raw data.

### Participant recruitment, follow-up

Healthcare workers (physicians, nurses, nursing assistant, and other essential workers) in direct contact with patients during the first, second or third wave of COVID-19 were recruited between 3 March 2020 and 22 March 2021. Inclusion criteria were agreeing to participate and confirming their availability for the follow-up sessions 7, 15, 30, 60, 90, 180, 270, and 360, and 450 days and, 18, 24,30, and 36 months after their first visit (baseline). The first follow-up was on 5 May 2020 and the last on 11 November 2021. Participants with less than two visits were considered to be a drop-out. At baseline, participants were categorized to their groups (infected or uninfected) based on RT-PCR, as well as IgM(N) and IgG(N) antibody tests: If any of the two tests was positive, the participant was considered infected. If an uninfected participant got infected, they started again the study as an infected participant, repeating the serological tests on the days said before specified above. The analyses reported in this work included only participants with positive SARS-CoV-2 antibodies, as considered infected participants.

Participants completed several clinical questionnaires and were examined for COVID-19-specific symptoms during the baseline visit, when the RT-PCR test with nasal and oropharyngeal swab and the antibody tests were performed. The RT-PCR test was repeated at the second visit, 7 days after the baseline visit. The antibody tests were repeated at 15, 30, 60, 90, 180, 270, 360, and 450 days following the baseline visit. Day 0 is defined as day 0 from date of diagnosis. The analysis of IgG(S) levels stopped as participants were vaccinated, while the IgM(N) and IgG(N) continued. Infected participants were divided into three different disease severity subgroups according to their symptomology: (1) asymptomatic: no symptoms; (2) mild-moderate: people with one or more clinical symptoms characteristic of COVID-19 and who did not require hospital admission; (3) severe-critical: patients who required hospital admission. The characteristics of the whole ProHEpiC-19 cohort, including the healthy group, at baseline are described in Table1.

**Table 1:** Demographics and PCR testing for the participants according to their disease severity

	Negative at baseline and during follow-up N = 295 (38.4)	Asymptomatic N = 73 (9.5)	Mild-moderate illness N = 363 (47.2)	Severe-critical illness N = 38 (4.9)	Total N = 769
Age (years)	46 (38.5–56) [19–66]	45 (31–52) [19–66]	45 (35–53) [18–66]	56.5 (50–61) [30–66]	46 (36–54) [18–66]
Gender assigned at birth					
Female	238 (80.7)	48 (65.7)	274 (75.5)	18 (47.4)	578 (75.2)
Male	57 (19.3)	25 (34.3)	89 (24.5)	20 (52.6)	191 (24.8)
Profession					
Doctor	112 (38.0)	9 (12.3)	76 (20.9)	13 (34.2)	210 (27.3)
Nurse	95 (32.2)	19 (26.0)	96 (26.4)	10 (26.3)	220 (28.6)
Nurse assistant	13 (4.41)	9 (12.3)	34 (9.37)	4 (10.5)	60 (7.80)
Others	75 (25.4)	36 (49.3)	157 (43.3)	11 (28.9)	279 (36.3)
Highest educational level attained					
Higher level vocational school	34 (11.8)	6 (8.57)	32 (9.22)	1 (2.78)	73 (9.84)
University	27 (9.34)	29 (41.4)	94 (27.1)	10 (27.8)	160 (21.6)
Others	228 (78.9)	35 (50.0)	221 (63.7)	25 (69.4)	509 (68.6)
NA	6	3	16	2	27
Marital status					
Single	44 (15.3)	16 (25.0)	63 (18.2)	3 (8.33)	126 (17.1)
Married/cohabitation	196 (68.1)	42 (65.6)	255 (73.5)	29 (80.6)	522 (71.0)
Divorced	43 (14.9)	3 (4.69)	22 (6.34)	3 (8.33)	71 (9.66)
Widow	5 (1.74)	3 (4.69)	7 (2.02)	1 (2.78)	16 (2.18)
NA	7	9	16	2	34
Nationality					
Spain	270 (95.4)	57 (90.5)	291 (87.7)	35 (100)	653 (91.6)
European Union	1 (0.35)	0 (0.00)	1 (0.30)	0 (0.00)	2 (0.28)
South America	7 (2.47)	1 (1.59)	23 (6.93)	0 (0.00)	31 (4.35)
Others	5 (1.77)	5 (7.94)	17 (5.12)	0 (0.00)	27 (3.79)
NA	12	10	31	3	59
Number of symptoms at baseline	–	0 (0) [0–0]	7 (4–9) [1–17]	9 (8–11) [2–17]	6 (2–9) [0–17] (*)
Days of follow-up	424 (269.5–481) [13–545]	302 (189–365) [14–527]	350 (271–434.5) [13–537]	438.5 (357.8–475.5) [156–505]	363 (269–464) [13–545]
Days since first positive diagnosis test	–	302 (220.5–374.5) [14–602]	365 (287–429) [28–617]	492 (341.8–540.8) [28–582]	370 (286–510) (*) [14–617]
≥ 1 positive PCR during follow-up	0 (0)	20 (27.4)	118 (32.5)	2 (5.3)	204 (26.5)

Categoric variables are described as N (%), and numeric variables as median (IQR) [min, max]

*NA* not available

In categories with NA, percentages were calculated excluding these answers. Diagnosis was made based on a positive PCR or IgM(N) or IgG(N) test. *Excludes negative participants. “Others” category in “Profession” includes physiotherapists, management and administration staff, and social workers; “Others” category in “Highest educational level attained” includes primary (5–12 years old) and secondary education (12–16 years old); and “Others” category in “Nationality” includes participants from Morocco, Senegal and Russia

### SARS-CoV-2 detection and quantification of IgM and IgG

RT-PCR was used as the primary diagnostic test. RNA for RT-PCR testing was extracted from fresh samples using the STARMag 2019-nCoV kit by means of a liquid-dispensing robot. RNA detection was performed using the Allplex SARS-CoV-2 assay, a multiplex RT-PCR assay able to detect four SARS-CoV-2 target genes in a single tube. For the antibody tests, we conducted a pre-validation study with six different commercially available and IVD-CE-approved ELISA tests and selected an anti-SARS-CoV-2 IgG(N) and IgM(N) enzyme immunoassay (ELISA) kits based on their performance. Infected participants were also tested for and measured the level of antibodies against the spike (S) subunit of SARS-CoV-2 by means of the DECOV1901 ELISA kit, which allows quantitative measurement of IgG. Please, see study protocol NCT04885478 (also attached as Additional file 1) to more details.

### Data sources

We used two data sources: (1) A database has been created for the ProHEpiC-19 cohort. The database is stored, curated, and validated in a centralized data warehouse at the Catalan Health Institute, and it is suitable to manage the demographic, social, and clinical data of professionals; (2) results of the SARS-COV-2 antibody tests; (3) results of the RT-PCR test. (4) We also implemented a Teleform-based data collection specific for the study, which includes the unique numeric patient identifier and the results of the serological and immunological tests. The unique numeric patient identifier allows to link the two data sources.

### Sample size

Sample size and power estimations can be found in the supplementary protocol. The one-way ANOVA of the total collected sample of 478 infected participants (72, 367, and 39 from the three subgroups) was estimated to achieve a 100% power to detect differences among the means versus the alternative of equal means using an F test with a 0.05 significance level and an effect size of η^2^ = 0.06.

### Statistical analysis

Missing values were found only in in the following sociodemographic variables: education (3.5%), marital status (4.6%), and nationality (7.5%).

The evolution of antibody test was considered both as a categorical (i.e. positive/negative result) and as a continuous scalar variable. Descriptive analyses were performed to characterise the immune response at each timepoints with the occurrence and frequency for categorical variables and median and inter-quartile range for numeric variables with non-normal distributions confirmed. The antibody levels were analysed with respect to time, disease severity, and gender. We stratified antibody levels by days since diagnosis, describing them by boxplots and comparing the timepoints using a Kruskal–Wallis test, followed by Holm-adjusted Dunn’s test. Then, at each timepoint, differences were assessed according to disease severity (Kruskal–Wallis followed by Holm-adjusted Dunn's test) and (Mann–Whitney *U* test). For a more detailed description of the antibody kinetics, we also fitted locally estimated scatterplot smoothing models (LOESS) and calculated their 95% confidence intervals (CIs). LOESS is an exploratory, nonparametric technique that is flexible, fast and easily deployed to determine if the evolution of the antibody levels is linear or non-linear. LOESS is useful for exploration but it does not provide a regression equation describing the evolution, neither does LOESS provides regression coefficients and p-values for making inferences and comparisons between groups. After LOESS indicated that the antibody evolution was non-linear and because the antibody levels were repeated measures from different time points, it is necessary to fit a regression model using a nonlinear mixed-effects model (NLME). Finally, nonlinear mixed effects models (NLME) were also fitted to obtain the curves characterising the antibody kinetics. In these models, each parameter was assumed to have a fixed and a random effect. Both LOESS and NLME models were first fitted on data from all patients and then on data stratified by disease severity and gender. Model diagnostics were performed based on residual analysis, and goodness-of-fit was checked with Akaike and Bayesian Information Criteria (AIC and BIC). For these all analyses except LOESS, time from diagnosis was discretised into time bins so that tests performed between the designated time points were considered as performed at the earlier time point. Therefore, tests performed in the first 14 days since diagnosis are treated as “Day 0” and tests performed between day 450 and 615 (i.e. the last day observed, see Table 1) are treated as “Day 450”. For the complete list and description of the time bins, see Additional file 1: Table S1.

All tests were two-sided, and statistical probability of p < 0.05 was considered significant. All analyses were performed using R version 4.0.4. See Additional file 1 for more information about the statistical methods.

## Results

### Participant characteristics

A total of 860 participants were recruited, of whom 769 were eligible; 443 (57.6%) tested positive at baseline, and 31 (4%) had their first positive SARS-CoV-2 antibody test during follow-up (Additional file 1: Fig. S1, Table 1). Women had more often asymptomatic or mild disease, while men tended to have more severe disease (p-value = 0.001). Differences in the prevalence of specific clinical symptoms according to disease severity and gender are shown in Additional file 1: Table S2.

### Seroprevalence

Table 2A presents the frequency of each possible combination of antibody test results at each time-point. At baseline, more than one third of the participants with available data (38.7%) tested negative for all antibodies, but this proportion decreased over time. From day 15 to day 180 of infection, more than 45% of participants tested positive for all antibodies. However, this proportion also decreased from day 180 as the number of participants with positive IgM(N) values fell. By day 270, 11.8% of the participants were negative for all antibody tests, while 88.2% of the participants were positive for at least IgG(N) or IgG(S). As Table 2B shows, 68.2% of participants still had IgG(N) values over the positivity threshold at day 360.

**Table 2:**
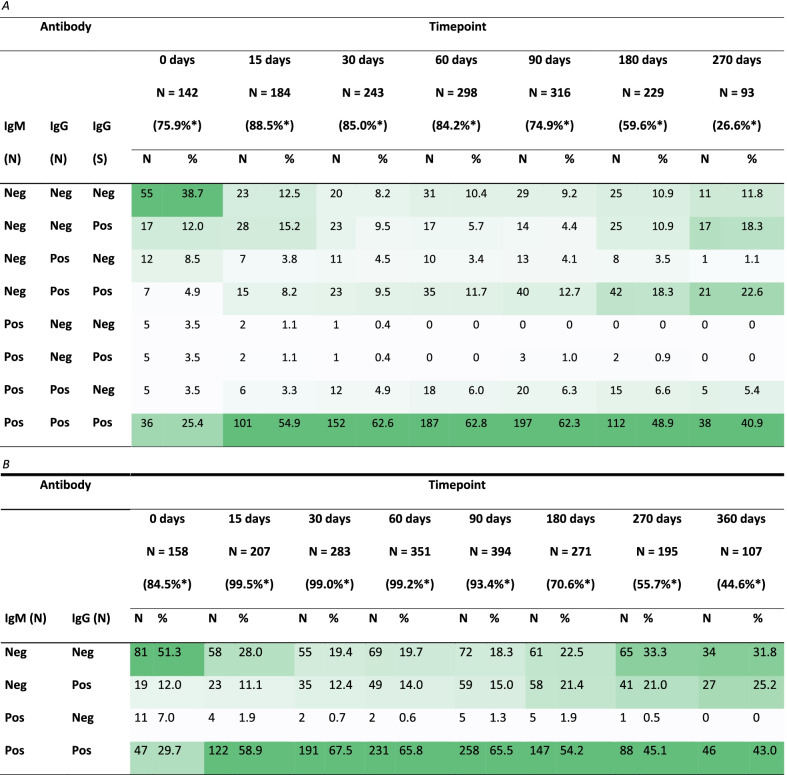
Description (N, %) of the results of the antibodies tests

Each column shows the participants that had one of the combinations of the diagnostic test results at each timepoint. Column shading highlights the most frequent results combinations at each timepoint. For Table 2A, only the records with available results for all three antibodies were used, while for Table 2B all records including (N) antibodies were used. *These percentages have been calculated using the maximum number of available samples per timepoint (i.e. (N) samples). This information is available at Table

### Levels of SARS-CoV-2 antibodies stratified by gender and disease severity

We found a statistically significant difference in all antibody levels across clinical conditions (p < 0.001 both overall and pairwise comparisons). There was also a significant difference in antibody levels between men and women for all immunoglobulins: IgM(N) (p = 0.01), IgG(N) (p < 0.001), and IgG(S) (p = 0.006).

**Fig. 1:**
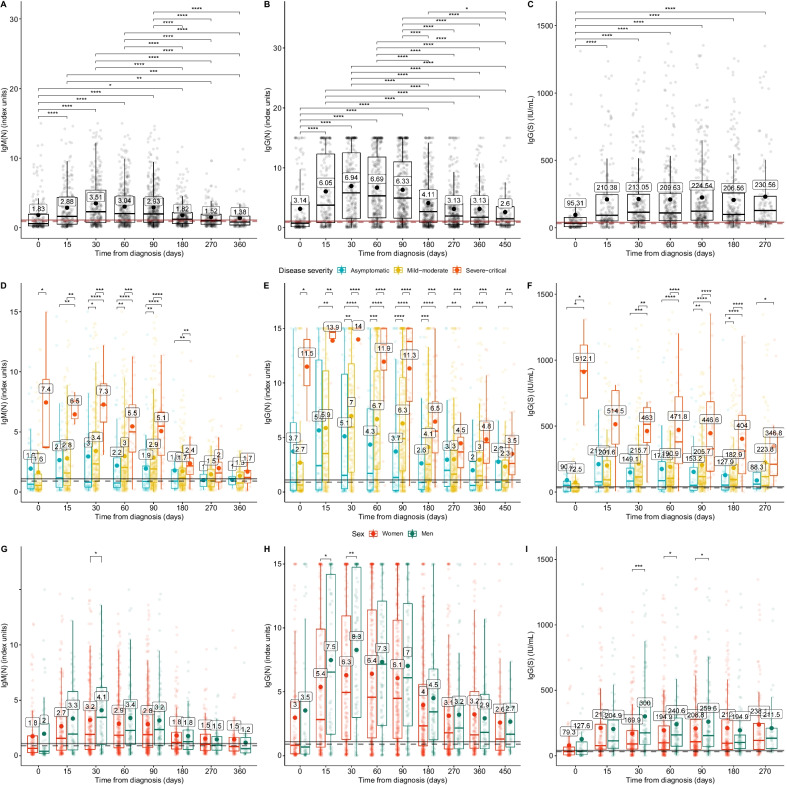
IgM (N), IgG (N) ang IgG(S) levels, by days since diagnosis. Antibody levels are represented with a boxplot together with a dot and text describing their mean value. The dashed and the solid horizontal lines represent the uncertainty and positivity thresholds, respectively. **A**–**C** show the significant differences in the median antibody levels between days. **D**–**F** show significant differences in the median antibody levels across disease severity at each timepoint. Finally, **G**–**I** show the significant differences in antibody levels between genders at each timepoint. Significance levels were reported as: * for p-value ≤ 0.05; ** for p-value ≤ 0.01; *** for p-value ≤ 0.001; and **** for p-value ≤ 0.0001

Figure 1 shows the differences in antibody levels between the different time-points. Pairwise comparisons show that both IgM(N) and IgG(N) levels from day 270 are no longer significantly different from those on day 0 (Figs. 1A-B). Both IgM(N) and IgG(N) levels present a rise until and fall from day 30 (Fig. 1A-B), while IgG(S) levels remain relatively constant following the first rise at day 15 (Fig. 1C). All antibody levels of patients with severe-critical disease were the highest up to day 180 (IgM(N)), day 450 (IgG(N) and day 270 (IgG(S)) (Fig. 1D-F). Resulting p-values are shown in the figure. Median IgM(N) levels were below the positivity threshold in people with asymptomatic and mild-moderate diseases after day 270 from diagnosis (Fig. 1D). However, IgG(N, S) levels still surpassed this threshold at day 360 (Fig. 1E-F). In terms of gender, men showed higher levels than women at day 30 for all antibodies (Fig. 1G-I) and days 60 and 90 for IgG(S) (Fig. 1I).

### Kinetics of IgM and IgG

**Fig. 2:**
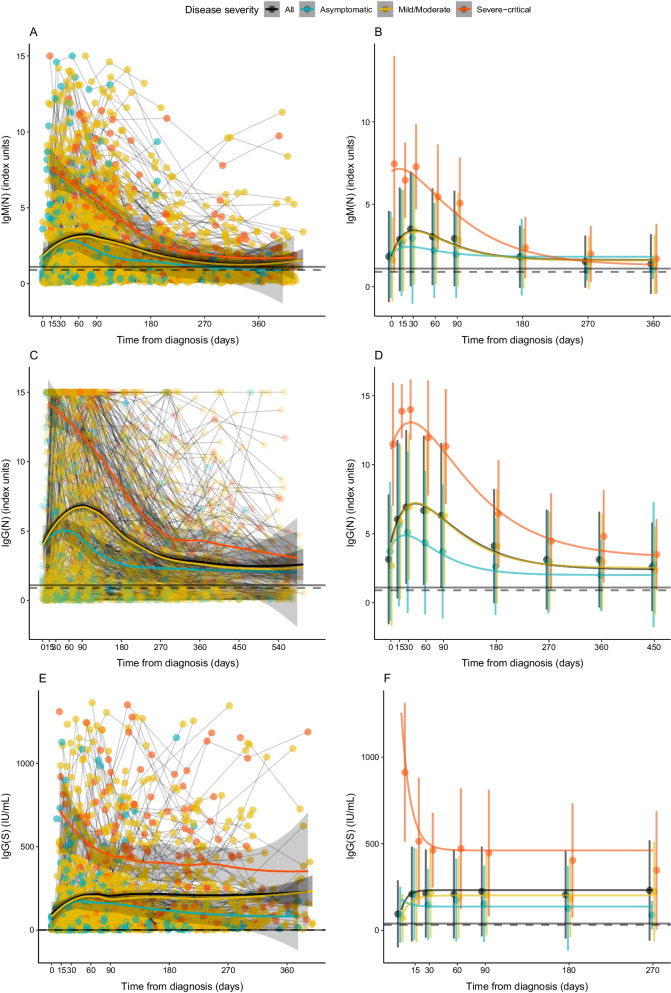
Kinetics of IgM(N), IgG(N) and IgG(S) levels since diagnosis in the total sample and stratified by disease severity. **A**, **C** and **E** show the LOESS regression models, connecting datapoints belonging to the same participants. **B**, **D** and **F** show the estimated non-linear mixed-effect (NLME) model curves. Each point corresponds to the mean value at each time point. The bars correspond to the standard deviation. The dashed and the solid horizontal lines represent the uncertainty and positivity thresholds, respectively

IgG(N) and IgG(S) had a different kinetics. In relation to the kinetics of the three antibodies stratified by disease severity, both LOESS (Fig. 2A, C, E) and NLME (Fig. 2B, D, F) showed a general rise in both IgM(N) and IgG(N) levels up to day 30 followed by a decay, and both the height of the peak and the rate of decay depended on disease severity. IgG(S) levels increased at day 15 and remained relatively constant over time.

Additional file 1: Table S3 shows the parameters estimated for each component of the NLME curves. Q-Q plots of each model are available in Additional file 1: Fig. S2. For both IgM(N) and IgG(N), initial levels were significantly higher in severe-critical. IgM(N) levels from asymptomatic participants decreased quicker, while those of severe-critical decreased slower than mild-moderate participants. This slower decline also occurs in IgG(N). Similarly to previous analyses, levels of IgG(S) increased with disease severity for all timepoints, and the levels were practically constant from day 30 in all groups.

**Fig. 3:**
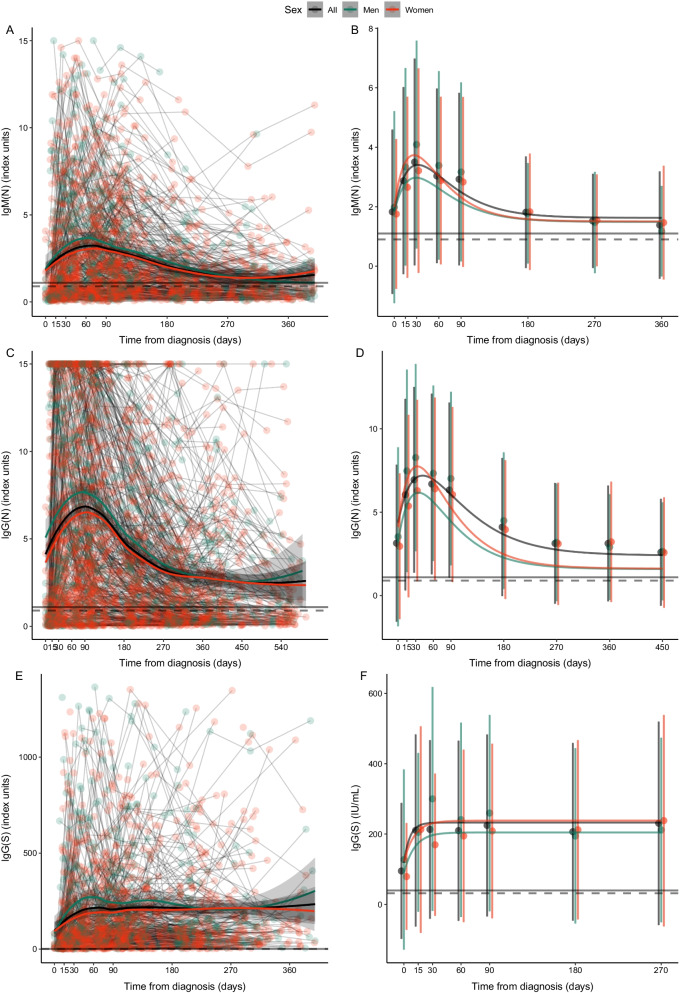
Kinetics of IgM(N), IgG(N) and IgG(S) levels since diagnosis in the total sample and stratified by gender. **A**, **C** and **E** show the LOESS regression models, connecting datapoints belonging to the same participants. **B**, **D** and **F** show the estimated non-linear mixed-effect (NLME) model curves. Each point corresponds to the mean value at each time point. The bars correspond to the standard deviation. The dashed and the solid horizontal lines represent the uncertainty and positivity thresholds, respectively

Figure 3 demonstrates the kinetics stratified by gender. The evolution patterns of the three antibodies are practically identical except for the peak in men on day 30. Correspondingly, the initial increase rates are also higher in men. The parameters of these NLME curves can be also found in Additional file 1: Table S3.

## Discussion

Our study draws strength from using longitudinal data from a large, representative, well characterized, and stratified sample of healthcare workers. The immune response to SARS-CoV-2 differs across disease severity. Our results show that antibody response starts within 15 days of the infection for the three studied isotypes. Thereafter, their behaviour diverges according to disease severity. We found a higher level of antibodies in patients with severe versus asymptomatic infections [11]. Our results also corroborate the early appearance of IgG(N) and (S) [6, 14]

Asymptomatic individuals maintain antibody levels above the threshold for IgM(N) up to 180 days, while antibodies for IgG(N) and (S) remain above the positivity threshold until day 270. The long-term presence of IgG(N) antibodies and their protective efficacy, however, needs further research [15, 16] Compared with asymptomatic cases, participants with mild-moderate COVID-19 presented higher IgG antibody levels, and their IgM(N) levels also remained above the threshold for the entire follow-up. These findings are similar to those reported at 180 days in a longitudinal study of IgM(S) and can be explained by the differentiation of B cells to IgM memory plasma cells that continue to produce the IgM isotype antibodies for at least a year [17]. Another recent study also found positive IgM levels for up to one year, although the isotypes IgM(S) rather than IgM(N). Severe-critical participants had higher antibody levels than the other groups in the first 30 days of follow-up and maintained the highest levels for all three isotypes throughout the entire follow-up period. This is consistent with the results of other studies conducted over a shorter period of time [9, 18]

The LOESS models describe the trajectories of the antibody levels, while the NLME allows to compare the stratified trajectories while accounting for the non-linearity of the antibody level kinetics. Our study further elaborates the kinetics of the humoral immune response while appropriately accounting for its characteristics and influencing factors as justified by the model selection. Our results are better equipped to inform social distancing and (re)vaccination policies. Kinetic antibodies results are critically important in the design and implementation of epidemiological models and public health measures such as social distancing policy and vaccination models. However, additional information regarding the value of the antibody kinetics, and protection from infection or disease severity should increment the impact for decision making in public health policies. Furthermore, our analyses can be easily adapted for estimating antibody levels measured in other units by applying the corresponding conversion factors. Likewise, they can be also employed to investigate the kinetics after vaccination, thus assessing the effectiveness of the vaccines in comparison with the immunisation due to infection and informing the criteria for revaccination.

To date, our study provides the most detailed and comparative description of the kinetics of IgG(N) and IgG(S) production after SARS-CoV-2 infection. IgG(N) levels show a rapid rise followed by a decay with a slope depending on disease severity, and it stabilizes between day 270 and 360. In contrast, IgG(S) kinetics shows a plateau from day 30 post-infection. This difference has also been reported in another study [19] and may be attributable to the differences between the S and N proteins of SARS-COV-2 in their molecular structure, their abundance in the viral particle, and their specific functions. While the former facilitates the entry of the virus into the host cell, the latter has a role in viral genomic packaging [20]. Monitoring of both IgG N and S antibodies can help identify stimulation of memory cells triggered by the two different antigens. Turner et al. [17] analysed the frequency of S-specific plasma cells in bone marrow of convalescent individuals, and observed that infection with SARS-CoV-2 is able to induce resting memory B cells and long-lived bone marrow plasma cells, supporting the existence of a robust humoral immune memory in infected individuals. Cohen KW et al. confirmed the former results and also analysed specific T-cells to SARS COV-2 [21]. Interestingly, they found that CD4 + T cell responses equally target several SARS-CoV-2 proteins, whereas CD8 + T cells preferentially target the N protein, highlighting the potential importance of including this antigen in future vaccines [21]. In addition, the fact that the IgG(N) response is persistent over time supports that this antigen could be monitored among other parameters (Specific T Cell and Neutralysing antibodies) to define subgroups of people who develop an efficient immune response [22–24].The maintenance of an IgG(N) and IgG(S) as well as specific T and B immune responses may protect against possible reinfections and render (re)vaccination redundant. Further in-depth immunological studies will be necessary to address their role in protection from severe cases—including death—, and in reinfection after both, infection and vaccination [25]

Humoral immune response seems to have a very similar trajectory in men and women, although we found higher IgG(N) or IgG(S) levels in men at all timepoints. Other studies with an eight-month follow-up indicate that gender and disease severity is associated due to differences in immune memory to SARS-CoV-2. However, most of the heterogeneity in immune memory to SARS-CoV-2 is still unexplained, and further investigation on the role of cellular immunity is also needed, especially in vulnerable groups, (non-seroconvertors, people with immunodeficiencies, and autoimmune disorders) [2, 26]

Anti-SARS-CoV2 antibodies were determined by ELISA techniques. The maximum levels of IgM(N) and IgG(N) antibodies tested could be even higher than reported, as the technique used was semi-quantitative due to the limited availability of quantitative in vitro diagnostic techniques at the beginning of the pandemic. Although, the WHO’s international standard for quantification of IgG(S) antibodies was established after the study start, we retrospectively analysed the samples for IgG(S) using a quantitative technique. The follow-up of IgG(S) levels stopped as participants were vaccinated, while the IgM(N) and IgG(N) continued.

Interpretation of these results should consider the limitations of the study. First, the majority of the ProHEpiC-19 sample are of white European origin, so findings might not be generalizable to other ethnic groups. Second, differences due to gender or disease severity were considered in the analysis. In addition to these, the study could be extended to include many other factors such as age, nationality, socioeconomic status or comorbidities, but the homogeneity of the sample in these aspects did not allow sufficient statistical power to do so. Future work could broaden the sample recruitment to people more diverse in these respects to extend the generality of the findings.

As more and more people are getting vaccinated, it is important to understand whether and how the immunity due to vaccination is different from that due to the infection. Are the kinetics and the durations of the antibody levels similar? Epidemiological modelling studies, especially long-term immunity monitoring, are focusing on the SARS-CoV-2 nowadays, however, one must also evaluate the interactions between SARS-CoV-2 and other coronaviruses for potential cross-immunisation [25, 27]. Moreover, epidemiological modelling of SARS-CoV-2 can also benefit from that of other viruses, such as flu and HIV-1; and vice versa [28, 29]. Second, the code to specify and fit the NLME is available, and one can exploit and extend as they see fit, and the results of this study will inform the clinical practice guidelines to assess the SARS-COV-2 seroprevalence.

To conclude, we monitored three types of antibodies for 17 months, and analysed their levels and kinetics while also considering the effect of gender and disease severity. NLME models allowed a more detailed understanding of the trajectories, confirming that infected people can maintain immunity due to the prolonged seroprevalence of IgG isotypes. While our study focused on the healthcare workers due to their importance in fighting against the pandemic, larger studies with a more heterogeneous sample and longer follow-up period can provide better generalisability and potentially elaborate on further effects. Even though the fitted estimating equations for the evolution of each antibody levels are provided in Additional file 1, NMLE models should be considered as a method to study the temporal evolution, instead of a punctual estimator. Due to the standardised quantitative assessment of IgG(S), our kinetic models can serve as a reference for future studies and inform social distancing and vaccination strategies.

## Supplementary Information

**Additional file 1: Table S1.** Number of available samples of each SARS-CoV-2 antibody per assessment timepoint. **Table S2.** Description (N, %) of the main symptoms in participants according to disease severity and sex assigned at birth. **Table S3.** Parameter estimation for SARS-CoV-2 antibodies (IgM(N), IgG(N), IgG(S)) NLME models. **Figure S1.** Flow chart of the ProHEpiC-19 study participants, including the recruitment procedure and the type of relationship with Sars-CoV-2. The analysis considered the “positive at baseline or during follow-up” participants. **Figure S2.** Q-Q plots of the non-linear mixed-effects models.

## Data Availability

The datasets generated and/or analysed during the current study are not publicly available due to the privacy conditions to which participants agreed in the informed consent form, but are available from the corresponding author on reasonable request. However, synthetic data generated from the original data to reproduce the analysis can be found at: https://github.com/IDIAPJGol/ProHEpic_Antibodies.
