# Supplementary material for: Kinetics of humoral immune response over 17 months of COVID-19 pandemic in a large cohort of healthcare workers in Spain: the ProHEpiC-19 study
Source: BMC Infect Dis. 2022 Sep 3;22:721. doi: 10.1186/s12879-022-07696-6 (PMC9439943; doi:10.1186/s12879-022-07696-6)
Supplement: Supplementary file 1 — Additional file 1: Table S1. Number of available samples of each SARS-CoV-2 antibody per assessment timepoint. Table S2. Description (N, %) of the main symptoms in participants according to disease severity and sex assigned at birth. Table S3. Parameter estimation for SARS-CoV-2 antibodies (IgM(N), IgG(N), IgG(S)) NLME models. Figure S1. Flow chart of the ProHEpiC-19 study participants, including the recruitment procedure and the type of relationship with Sars-CoV-2. The analysis considered the “positive at baseline or during follow-up” participants. Figure S2. Q-Q plots of the non-linear mixed-effects models. [file 12879_2022_7696_MOESM1_ESM.docx]

Supplementary Material

index

[2 Participant recruitment 1](#_Toc105162975)

[3 Statistical analysis 2](#_Toc105162976)

[3.1 Statistical tests 2](#_Toc105162977)

[3.2 Initial analysis of the evolution of antibody levels from diagnosis 3](#_Toc105162978)

[3.3 Nonlinear mixed-effects (NLME) models 4](#_Toc105162979)

[4 References 10](#_Toc105162980)

[5 Supplementary tables 11](#_Toc105162981)

[6 Supplementary figures 16](#_Toc105162982)

# Participant recruitment

Participants recruited with negative tests who seroconverted during the follow-up period (N = 31 (4.0%)) were considered in the analysis of positive participants as if they had been recruited at the time of diagnosis. The temporal relationship between the time of diagnosis and the first study visit of the 474 SARS-CoV-2 positive participants is presented in the table below. The number of days from diagnosis was discretized as follows: diagnoses set in the first 14 days of the study were treated as "day 0"; diagnoses between days 15 and 29, as "day 15"; between days 30 and 59, as "day 30"; between days 60 and 89, as "day 60"; between days 90 and 179, as "day 90"; between days 180 and 269, as "day 180"; between days 270 and 360, as "day 270"; and since the 360^th^ day until the end of study, as “day 360”.

| Timepoint  (days from diagnosis) | Patients with positive tests (N) | Frequency | Cumulative frequency |
| --- | --- | --- | --- |
| 0 | 161 | 34.0 | 34.0 |
| 15 | 76 | 16.0 | 50.0 |
| 30 | 64 | 13.5 | 63.5 |
| 60 | 103 | 21.7 | 85.2 |
| 90 | 48 | 10.1 | 95.4 |
| 180 | 19 | 4.0 | 99.4 |
| 270 | 3 | 0.6 | 100 |
| 360 | 0 | 0 | 100 |
| 450 | 0 | 0 | 100 |

# Statistical analysis

An outlier detection on IgG(S) antibody levels was performed, considering outliers the values belonging to the most extreme 0.3%, according to the z-score (i.e. z-score <-3 or >3) after scaling the variable. Twenty-seven IgG(S) values were thus excluded. No outlier detection was performed on IgM(N) and IgG(N) antibodies as their value was capped ([0, 15]).

All tests were two-sided, and statistical probability of p < 0.05 was considered significant. All analyses were performed using R version 4.0.4 or higher. The code that produced all the results can be found at <https://github.com/IDIAPJGol/ProHEpic_Antibodies>.

## Statistical tests

We used chi-squared test to identify significant interactions between the expected and the observed frequencies for categorical comparisons (e.g., the prevalence of symptoms according to biological sex or disease severity).

To evaluate differences in SARS-CoV-2 antibody (IgM, IgG) levels across the clinical spectrum, we used a Kruskal-Wallis test after assessing the normality of the distribution (Shapiro-test p-value < 0.05). A post-hoc analysis was performed using a Holm-adjusted Dunn’s test to find significant differences. Similarly, a Holm-adjusted Mann-Whitney test was performed to compare the antibody levels of men and women. These first tests were performed using all available samples, regardless of the timepoint in which they were collected.

## Initial analysis of the evolution of antibody levels from diagnosis

The evolution of antibodies levels since diagnosis was visualized by means of boxplots and analyzed by means of statistical tests. Time since diagnosis was discretized into time bins similarly to the time of diagnosis as described in Section 1. The number of available samples at each time bin and type of antibody is presented in Table S1.

Due to the very low proportion of participants arriving at day 360 of follow-up without being vaccinated and the outlier removal, the number of available samples of IgG(S) antibody was very low; therefore, they were excluded from further analyses.

Once the time was discretized, boxplots showing the distribution of the IgM and IgG (both N and S) values for each time bin were obtained. The overall distributions at each time bin were compared to find significant differences across time using a Holm-adjusted Dunn’s test. Stratified distributions at each time bin were analyzed to investigate the effect of sex and disease severity using a Holm-adjusted Mann-Whitney test and a Holm-adjusted Dunn’s test, respectively.

Locally estimated scatterplot smoothing (LOESS) models were fit to study the evolution of both IgM and IgG antibodies. LOESS regression consists of fitting simple models (e.g., first- or second-degree polynomials) in subsets of data determined by a nearest-neighbours algorithm. Figures 3 and S1 show the LOESS curves, stratified either by disease severity or sex, along with their 95% confidence interval (CI). We used all the data available except for participants who never tested positive, grouping the data points per participant in the figures.

## Nonlinear mixed-effects (NLME) models

Mixed-effects models are generally useful when there are multiple measures per participant, as in this study. Random-effects allow to generalise the results from a random sample to the whole population from which the sample has been drawn. It compares the variance explained by the modelled effect(s) to that of that due to the random sampling. The nonlinear models typically more flexible and able to describe more complex patterns in the data than their linear counterparts. Therefore, nonlinear models provide the best approach to characterise our cohort.

In this study, we used NLME models to investigate the kinetics of SARS-CoV-2 antibody (IgM, IgG) levels since the onset, as well as how the kinetics varies according to the disease severity and sex. “Mild to moderate illness” and “Women” were used as reference groups, respectively, as they were the largest groups. To fit these models, we used all the data available except for those from participants who never tested positive. Time from diagnosis was discretized as described in Section 1.

The general equations to be fitted to model mean values of antibodies at a time ***t*** was:

$$IgM\left( N \right) \left( t \right)=b_{1}+\left( b_{0}-b_{1} \right)e^{-k_{1}t}-b_{1}k_{1}(e^{-k_{1}t}-e^{-k_{2}t})$$

$$IgG\left( N \right) \left( t \right)=b_{1}+\left( b_{0}-b_{1} \right)e^{-k_{1}t}-b_{1}k_{1}(e^{-k_{1}t}-e^{-k_{2}t})$$

$$IgG\left( S \right) \left( t \right)=b_{1}+\left( b_{0}-b_{1} \right)e^{-k_{1}t}-b_{1}k_{1}e^{-k_{1}t}$$

*t* has been normalized according to the maximum length of follow-up (i.e., 360 for IgM(N), 450 for IgG(N), and 270 for IgG(S) antibody). This general equation corresponds to an equation describing an exponential rise followed by an exponential decay, where $b_{0}$ corresponds to the baseline value, $b_{1}$ to the asymptotic value (i.e., antibody levels at $t \to\infty$), $k_{1}$ to the rise rate, and $k_{2}$ to the decay rate.

Nonlinear mixed-effects models were specified and estimated using the nlme package (Pinheiro J. et al., 2020). This package needs to have initial values for the parameters, which were estimated using the mean values in each time point. The following table shows the initial values used to start the models. The final values of the parameters after fitting the model can be seen in Table S3. Q-Q plots of each model are available in Figure S2.

| **SARS-CoV-2 antibody** | **Model** | **Parameter** | **Intitial value** |
| --- | --- | --- | --- |
| **IgM (N)** | **Overall** | b0 - Baseline value | 2 |
|  |  | b1 - Asymptotic value (i.e., antibody levels at t →∞) | 1 |
|  |  | k_1 -_ Rise rate | 6 |
|  |  | k_2_ - Decay rate | 12 |
|  | **IgM (N)**  **~**  **Disease severity** | b_0_ (Mild-moderate) | 2 |
|  |  | b_0_ (Asymptomatic) | 0 |
|  |  | b_0_ (Severe-critical) | 0 |
|  |  | b_1_ (Mild-moderate) | 1 |
|  |  | b_1_ (Asymptomatic) | 0 |
|  |  | b_1_ (Severe-critical) | 0 |
|  |  | k_1_ (Mild-moderate) | 6 |
|  |  | k_1_ (Asymptomatic) | 0 |
|  |  | k_1_ (Severe/critical) | 0 |
|  |  | k_2_ (Mild-moderate) | 12 |
|  |  | k_2_ (Asymptomatic) | 0 |
|  |  | k_2_ (Severe-critical) | 0 |
|  | **IgM (N)**  **~**  **Gender** | b_0_ (Women) | 1.7 |
|  |  | b_0_ (Men) | 0 |
|  |  | b_1_ (Women) | 1.4 |
|  |  | b_1_ (Men) | 0 |
|  |  | k_1_ (Women) | 12 |
|  |  | k_1_ (Men) | 0 |
|  |  | k_2_ (Women) | 6 |
|  |  | k_2_ (Men) | 0 |
| **IgG (N)** | **Overall** | b0 - Baseline value | 3 |
|  |  | b1 - Asymptotic value (i.e., antibody levels at t →∞) | 2.6 |
|  |  | k_1 -_ Rise rate | 12 |
|  |  | k_2_ - Decay rate | 6 |
|  | **IgG (N)**  **~**  **Disease severity** | b_0_ (Mild-moderate) | 3.2 |
|  |  | b_0_ (Asymptomatic) | 0 |
|  |  | b_0_ (Severe-critical) | 0 |
|  |  | b_1_ (Mild-moderate) | 2.5 |
|  |  | b_1_ (Asymptomatic) | 0 |
|  |  | b_1_ (Severe-critical) | 0 |
|  |  | k_1_ (Mild-moderate) | 12 |
|  |  | k_1_ (Asymptomatic) | 0 |
|  |  | k_1_ (Severe-critical) | 0 |
|  |  | k_2_ (Mild-moderate) | 6 |
|  |  | k_2_ (Asymptomatic) | 0 |
|  |  | k_2_ (Severe-critical) | 0 |
|  | **IgG (N)**  **~**  **Gender** | b_0_ (Women) | 3 |
|  |  | b_0_ (Men) | 0 |
|  |  | b_1_ (Women) | 2.6 |
|  |  | b_1_ (Men) | 0 |
|  |  | k_1_ (Women) | 12 |
|  |  | k_1_ (Men) | 0 |
|  |  | k_2_ (Women) | 3 |
|  |  | k_2_ (Men) | 0 |
| **IgG (S)** | **Overall** | b0 - Baseline value | 90 |
|  |  | b1 - Asymptotic value (i.e., antibody levels at t →∞) | 210 |
|  |  | k_1 -_ Rise rate | 25 |
|  | **IgG (S)**  **~**  **Disease severity** | b_0_ (Mild-moderate) | 70 |
|  |  | b_0_ (Asymptomatic) | 20 |
|  |  | b_0_ (Severe-critical) | 840 |
|  |  | b_1_ (Mild-moderate) | 220 |
|  |  | b_1_ (Asymptomatic) | 0 |
|  |  | b_1_ (Severe-critical) | 0 |
|  |  | k_1_ (Mild-moderate) | 30 |
|  |  | k_1_ (Asymptomatic) | 0 |
|  |  | k_1_ (Severe-critical) | 0 |
|  | **IgG (S)**  **~**  **Gender** | b_0_ (Women) | 80 |
|  |  | b_0_ (Men) | 50 |
|  |  | b_1_ (Women) | 240 |
|  |  | b_1_ (Men) | 0 |
|  |  | k_1_ (Women) | 24 |
|  |  | k_1_ (Men) | 0 |

The adjusted **general** equations are:

$$IgM\left( N \right) \left( t \right)=1.63+0.23e^{-12.06t}-19.66(e^{-12.06t}-e^{-9.52t})$$

$$IgG\left( N \right) \left( t \right)=2.41+1.97e^{-10.85t}-26.15(e^{-10.85t}-e^{-7.03t})$$

$$IgG\left( S \right) \left( t \right)=232.55+9971.59e^{-43.37t}-10085.70e^{-43.37t}$$

Normalizing *t* according to the maximum length of follow-up (i.e., 360 for IgM(N), 450 for IgG(N), and 270 for IgG(S) antibody).

The adjusted equations for patients having **asymptomatic disease** are:

$$IgM\left( N \right) \left( t \right)=1.82+0.16e^{-13.61t}-24.77(e^{-13.61t}-e^{-12.81t})$$

$$IgG\left( N \right) \left( t \right)=2.01+2.03e^{-12.77t}-25.67(e^{-12.77t}-e^{-10.27t})$$

$$IgG\left( S \right) \left( t \right)=137.79+4299.36e^{-30.89t}-4256.33e^{-30.89t}$$

Normalizing *t* according to the maximum length of follow-up (i.e., 360 for IgM(N), 450 for IgG(N), and 270 for IgG(S) antibody).

The adjusted equations for patients with **mild to moderate disease** are:

$$IgM\left( N \right) \left( t \right)=1.6+0.03e^{-12.26t}-19.62(e^{-12.26t}-e^{-9.61t})$$

$$IgG\left( N \right) \left( t \right)=2.52+1.11e^{-11.63t}-29.31(e^{-11.63t}-e^{-97.73t})$$

$$IgG\left( S \right) \left( t \right)=202.37+5995.01e^{-30.25t}-6121.69e^{-30.25t}$$

Normalizing *t* according to the maximum length of follow-up (i.e., 360 for IgM(N), 450 for IgG(N), and 270 for IgG(S) antibody).

The adjusted equations for patients having a **severe to critical disease** are:

$$IgM\left( N \right) \left( t \right)=1.27+5.73e^{-10.60t}-13.46(e^{-10.60t}-e^{-5.21t})$$

$$IgG\left( N \right) \left( t \right)=3.25+7.88e^{-9.47t}-30.78(e^{-9.47t}-e^{-5.06t})$$

$$IgG\left( S \right) \left( t \right)=461.48+13635.77e^{-27.82t}-12838.37e^{-27.82t}$$

Normalizing *t* according to the maximum length of follow-up (i.e., 360 for IgM(N), 450 for IgG(N), and 270 for IgG(S) antibody).

The adjusted equations for **women** are:

$$IgM\left( N \right) \left( t \right)=1.49+0.31e^{-12.33t}-18.376(e^{-12.33t}-e^{-10.06t})$$

$$IgG\left( N \right) \left( t \right)=1.59+1.63e^{-13.96t}-22.20(e^{-13.96t}-e^{-8.41t})$$

$$IgG\left( S \right) \left( t \right)=204.47+4829.49e^{-24.18t}-4944.10e^{-24.18t}$$

Normalizing *t* according to the maximum length of follow-up (i.e., 360 for IgM(N), 450 for IgG(N), and 270 for IgG(S) antibody).

The adjusted equations for **men** are:

$$IgM\left( N \right) \left( t \right)=1.51+0.46e^{-14.12t}-21.32(e^{-14.12t}-e^{-10.84t})$$

$$IgG\left( N \right) \left( t \right)=1.62+2.49e^{-15.09t}-24.45(e^{-15.09t}-e^{-8.20t})$$

$$IgG\left( S \right) \left( t \right)=238.13+5696.27e^{-24.41t}-5812.75e^{-24.41t}$$

Normalizing *t* according to the maximum length of follow-up (i.e., 360 for IgM(N), 450 for IgG(N), and 270 for IgG(S) antibody).

# References

Pinheiro J, Bates D, DebRoy S, Sarkar D, R Core Team (2020). nlme: Linear and Nonlinear Mixed Effects Models. R package version 3.1-144. Available at: https://CRAN.R-project.org/package=nlme

# Supplementary tables

**Table S1.** Number of available samples of each SARS-CoV-2 antibody per assessment timepoint.

| **Timepoint   (days since diagnosis)** | **Mean number of days of follow-up** | **IgM(N) samples** | **IgG(N) samples** | **IgG(S) samples** |
| --- | --- | --- | --- | --- |
| **0** | 9.37 ± 4.04 | 187 | 161 | 142 |
| **15** | 22.8 ± 4.37 | 207 | 208 | 184 |
| **30** | 43.0 ± 7.87 | 283 | 286 | 244 |
| **60** | 74.5 ± 7.67 | 351 | 354 | 298 |
| **90** | 120 ± 22.7 | 394 | 422 | 319 |
| **180** | 215 ± 23.0 | 271 | 384 | 257 |
| **270** | 306 ± 23.8 | 195 | 350 | 113 |
| **360** | 397 ± 23.9 | 107 | 240 | - |
| **450** | 530 ± 39.3 | - | 138 | - |

*Notes:* Each timepoint represent a certain number of days since diagnosis.

**Table S2.** Description (N, %) of the main symptoms in participants according to disease severity and sex assigned at birth.

| **Symptom, n (%)** | **Mild-moderate illness N = 363 (47.2)** | | | | **Severe-critical illness N = 38 (4.9)** | | | | **P-value** |
| --- | --- | --- | --- | --- | --- | --- | --- | --- | --- |
|  | **Women N = 274 (75.5)** | **Men N = 89 (24.5)** | **P-value** | **Total** | **Women N = 18 (47.4)** | **Men N = 20 (52.6)** | **P-value** | **Total** |  |
| Headache | 191 (69.7) | 53 (59.6) | 0.10 | 246 (67.0) | 11 (61.1) | 12 (60.0) | 1.00 | 23 (59.0) | 0.52 |
| Diarrhea | 104 (38.0) | 30 (33.7) | 0.55 | 136 (37.1) | 9 (50.0) | 8 (40.0) | 0.77 | 17 (43.6) | 0.44 |
| Dyspnea | 62 (22.6) | 14 (15.7) | 0.22 | 75 (20.4) | 10 (55.6) | 9 (45.0) | 0.75 | 19 (48.7) | <0.001 |
| Fever | 138 (50.4) | 49 (55.1) | 0.52 | 192 (52.3) | 12 (66.7) | 19 (95.0) | 0.04 | 32 (82.1) | 0.001 |
| Cough | 152 (55.5) | 47 (52.8) | 0.75 | 200 (54.5) | 11 (61.1) | 10 (50.0) | 0.72 | 21 (53.8) | 1.00 |
| Anosmia | 143 (52.2) | 35 (39.3) | 0.05 | 183 (49.9) | 8 (44.4) | 7 (35.0) | 0.79 | 15 (38.5) | 0.34 |
| Arthralgias | 118 (43.1) | 25 (28.1) | 0.02 | 145 (39.5) | 10 (55.6) | 9 (45.0) | 0.75 | 20 (51.3) | 0.27 |
| Asthenia | 188 (68.6) | 47 (52.8) | 0.01 | 239 (65.1) | 15 (83.3) | 16 (80.0) | 1.00 | 31 (79.5) | 0.06 |
| Shivers | 94 (34.3) | 27 (30.3) | 0.58 | 124 (33.8) | 9 (50.0) | 7 (35.0) | 0.54 | 17 (43.6) | 0.37 |
| Chest pain | 41 (15.0) | 11 (12.4) | 0.66 | 51 (13.9) | 3 (16.7) | 2 (10.0) | 0.65 | 6 (15.4) | 1.00 |
| Epigastralgia | 42 (15.3) | 11 (12.4) | 0.61 | 54 (14.7) | 4 (22.2) | 5 (25.0) | 1.00 | 9 (23.1) | 0.22 |
| Discomfort | 160 (58.4) | 49 (55.1) | 0.67 | 210 (57.2) | 12 (66.7) | 15 (75.0) | 0.84 | 26 (66.7) | 0.15 |
| Myalgias | 143 (52.2) | 35 (39.3) | 0.05 | 179 (48.8) | 12 (66.7) | 15 (75.0) | 0.84 | 27 (69.2) | 0.02 |
| Nausea | 48 (17.5) | 10 (11.2) | 0.22 | 60 (16.3) | 4 (22.2) | 2 (10.0) | 0.40 | 6 (15.4) | 1.00 |
| Odinophagy | 75 (27.4) | 13 (14.6) | 0.02 | 87 (23.7) | 2 (11.1) | 0 (0.00) | 0.22 | 4 (10.3) | 0.01 |
| Congestion | 103 (37.6) | 24 (27.0) | 0.09 | 125 (34.1) | 2 (11.1) | 5 (25.0) | 0.41 | 6 (15.4) | 0.06 |
| Others | 70 (25.5) | 22 (24.7) | 0.99 | 92 (25.1) | 5 (27.8) | 2 (10.0) | 0.22 | 6 (15.4) | 0.45 |
| **Health service use, n (%)** |  |  |  |  |  |  |  |  |  |
| Emergency room | 52 (19.0) | 19 (21.3) | 0.74 | 70 (19.1) | 15 (83.3) | 18 (90.0) | 0.65 | 34 (87.2) | <0.001 |
| Hospital | 0 (0) | 0 (0) | - | 0 (0.00) | 18 (100) | 20 (100) | . | 39 (100) | <0.001 |
| ICU | 0 (0) | 0 (0) | - | 0 (0.00) | 1 (5.56) | 4 (20.0) | 0.34 | 6 (15.4) | <0.001 |

*Notes:* Prevalence of symptoms stratified by sex was also study in each clinical condition. The p-value column shows the corresponding Chi-square test result. Asymptomatic participants were excluded from this analysis as they did not report any symptom. Prevalence of disease severity is calculated on the total of infected participants.

**Table S3.** Parameter estimation for SARS-CoV-2 antibodies (IgM(N), IgG(N), IgG(S)) NLME models.

| **Model** | **Parameter** | **IgM (N)** | | | | | **IgG (N)** | | | | | **IgG (S)** | | | | |
| --- | --- | --- | --- | --- | --- | --- | --- | --- | --- | --- | --- | --- | --- | --- | --- | --- |
|  |  | **Variance of random effects residuals** | **BIC** | **AIC** | **Value (SE)** | **P-value** | **Variance of random effects residuals** | **BIC** | **AIC** | **Value (SE)** | **P-value** | **Variance of random effects residuals** | **BIC** | **AIC** | **Value (SE)** | **P-value** |
| Complete | b0 - Baseline value | 1.54 | 9310.09 | 9269.53 | 1.86 (0.12) | <0.001 | 4.51 | 12950.66 | 12909.69 | 4.38 (0.31) | <0.001 | 215 | 29105.5 | 29071.39 | 10204.14 (5998.61) | 0.09 |
|  | b1 - Asymptotic value (i.e., antibody levels at t →∞) |  |  |  | 1.63 (0.06) | <0.001 |  |  |  | 2.41 (0.22) | <0.001 |  |  |  | 232.55 (7.80) | <0.001 |
|  | k1 - Rise rate |  |  |  | 12.06 (0.28) | <0.001 |  |  |  | 10.85 (0.41) | <0.001 |  |  |  | 43.37 (26.25) | 0.1 |
|  | k2 - Decay rate |  |  |  | 9.52 (0.27) | <0.001 |  |  |  | 7.03 (0.47) | <0.001 |  |  |  | - | - |
| Antibody level ~ Disease severity | b0 (Mild-moderate) | 1.5 | 9272.91 | 9186.01 | 1.63 (0.14) | <0.001 | 4.37 | 12896.96 | 12809.18 | 3.63 (0.34) | <0.001 | 246 | 21089.66 | 21025.46 | 6197.38 (2622.84) | 0.02 |
|  | b0 (Asymptomatic) |  |  |  | 0.35 (0.26) | 0.18 |  |  |  | 0.41 (0.68) | 0.55 |  |  |  | -1760.23 (12739.14) | 0.89 |
|  | b0 (Severe-critical) |  |  |  | 5.37 (0.74) | <0.001 |  |  |  | 7.50 (1.77) | <0.001 |  |  |  | 7899.87 (5353.36) | 0.14 |
|  | b1 (Mild-moderate) |  |  |  | 1.60 (0.07) | <0.001 |  |  |  | 2.52 (0.23) | <0.001 |  |  |  | 202.37 (11.80) | <0.001 |
|  | b1 (Asymptomatic) |  |  |  | 0.22 (0.19) | 0.24 |  |  |  | -0.51 (0.58) | 0.38 |  |  |  | -64.58 (32.46) | 0.05 |
|  | b1 (Severe-critical) |  |  |  | -0.33 (0.24) | 0.17 |  |  |  | 0.73 (0.84) | 0.38 |  |  |  | 259.11 (35.82) | <0.001 |
|  | k1 (Mild-moderate) |  |  |  | 12.26 (0.32) | <0.001 |  |  |  | 11.63 (0.46) | <0.001 |  |  |  | 30.25 (13.44) | 0.03 |
|  | k1 (Asymptomatic) |  |  |  | 1.35 (0.87) | 0.12 |  |  |  | 1.14 (2.31) | 0.62 |  |  |  | 0.64 (88.30) | 0.99 |
|  | k1 (Severe-critical) |  |  |  | -1.66 (1.43) | 0.25 |  |  |  | -2.16 (1.13) | 0.06 |  |  |  | -2.43 (16.44) | 0.88 |
|  | k2 (Mild-moderate) |  |  |  | 9.61 (0.31) | <0.001 |  |  |  | 7.73 (0.50) | <0.001 |  |  |  | - | - |
|  | k2 (Asymptomatic) |  |  |  | 3.20 (0.89) | <0.001 |  |  |  | 2.54 (2.43) | 0.3 |  |  |  | - | - |
|  | k2 (Severe-critical) |  |  |  | -4.40 (0.77) | <0.001 |  |  |  | -2.67 (1.08) | 0.01 |  |  |  | - | - |
| Antibody level ~  Gender | b0 (Women) | 1.37 | 9445.64 | 9387.7 | 1.82 (0.13) | <0.001 | 2.49 | 13315.26 | 13256.74 | 3.22 (0.22) | <0.001 | 215 | 29585.77 | 29840.29 | 5033.96 (2317.42) | 0.03 |
|  | b0 (Men) |  |  |  | 0.17 (0.22) | 0.45 |  |  |  | 0.89 (0.39) | 0.02 |  |  |  | 900.44 (4571.75) | 0.84 |
|  | b1 (Women) |  |  |  | 1.49 (0.06) | <0.001 |  |  |  | 1.59 (0.08) | <0.001 |  |  |  | 204.47 (8.27) | <0.001 |
|  | b1 (Men) |  |  |  | 0.02 (0.11) | 0.86 |  |  |  | 0.03 (0.15) | 0.84 |  |  |  | 33.66 (14.75) | 0.02 |
|  | k1 (Women) |  |  |  | 12.33 (0.32) | <0.001 |  |  |  | 13.96 (0.46) | <0.001 |  |  |  | 24.18 (11.79) | 0.04 |
|  | k1 (Men) |  |  |  | 1.79 (0.57) | 0.002 |  |  |  | 1.13 (0.85) | 0.18 |  |  |  | 0.23 (20.82) | 0.99 |
|  | k2 (Women) |  |  |  | 10.06 (0.33) | <0.001 |  |  |  | 8.41 (0.33) | <0.001 |  |  |  | - | - |
|  | k2 (Men) |  |  |  | 0.78 (0.59) | 0.19 |  |  |  | -0.21 (0.60) | 0.73 |  |  |  | - | - |

AIC: Akaike information criterion; BIC: Bayesian information criterion; SE: standard error

*Notes:* Three different models were fitted for each antibody: the first one was general, the second one was stratified by clinical condition, and the third one was stratified by sex assigned at birth. The reference categories were mild-moderate and Women. The parameters of the other categories are expressed with reference to these categories. The fitted equations of these models can be found in section 2.3 of the Supplementary Methods.

# Supplementary figures


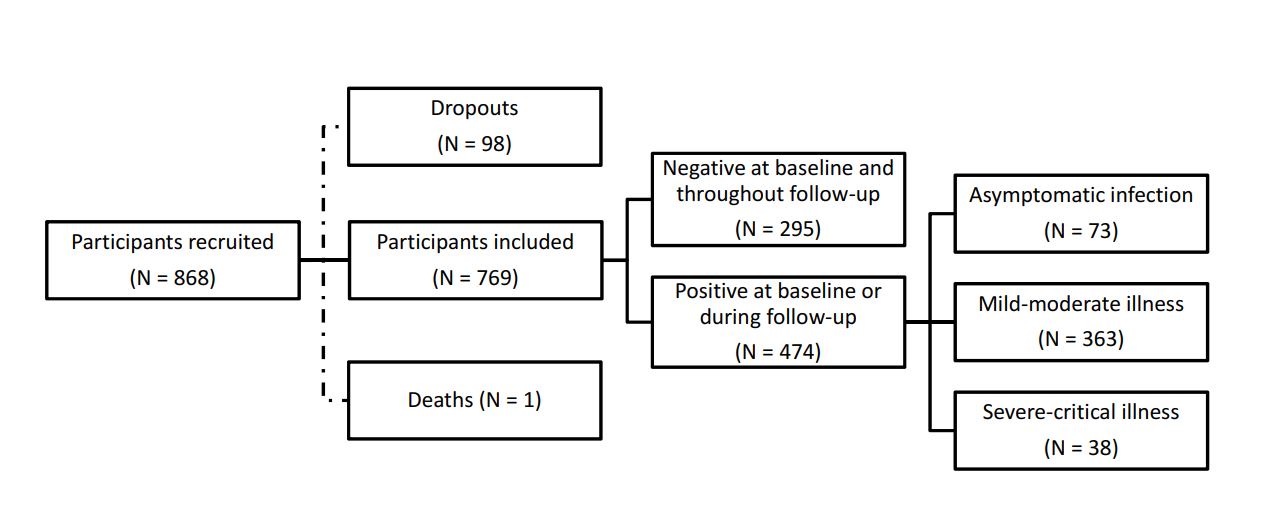
**Figure S1**. Flow chart of the ProHEpiC-19 study participants, including the recruitment procedure and the type of relationship with Sars-CoV-2. The analysis considered the “positive at baseline or during follow-up” participants.


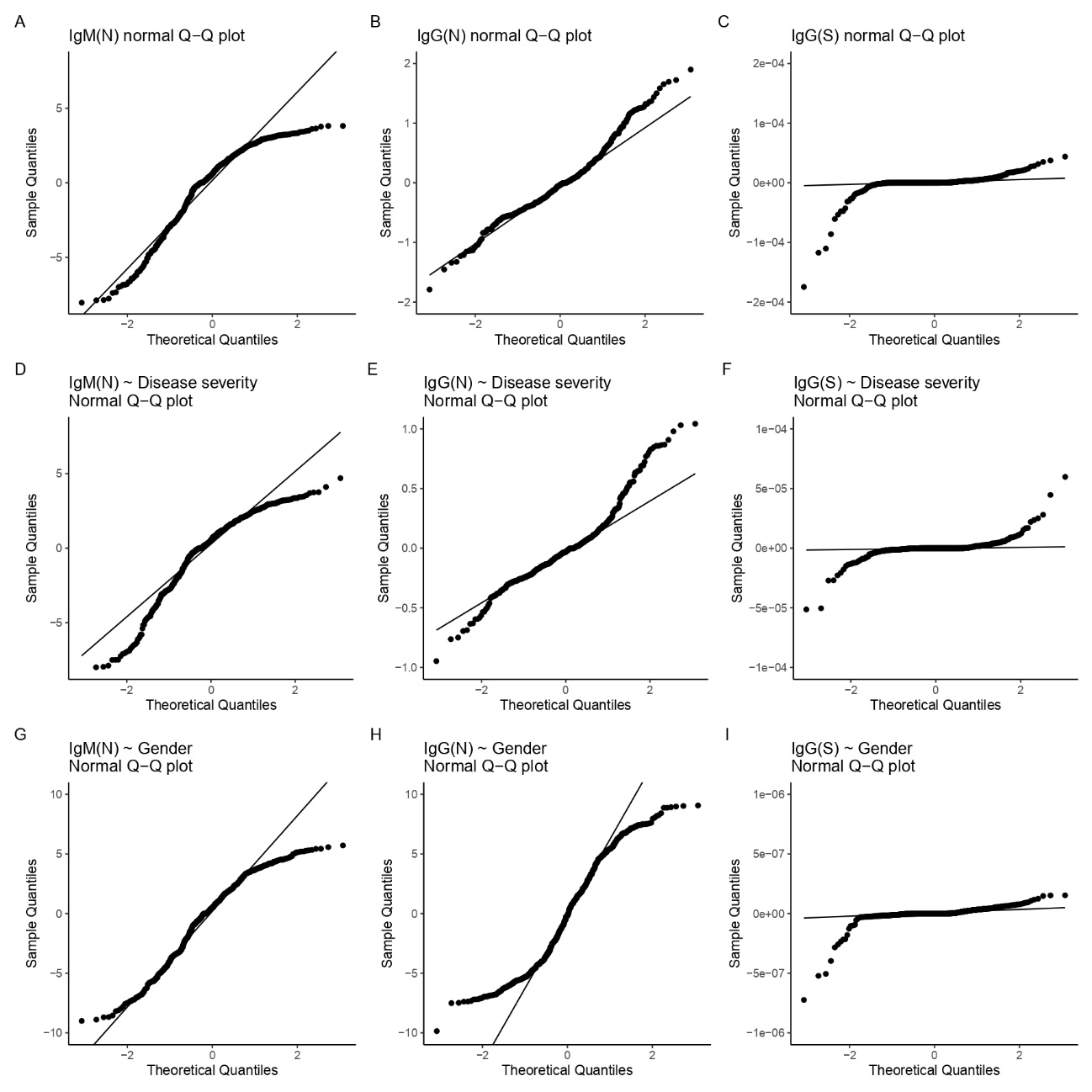


**Figure S2**. Q-Q plots of the non-linear mixed-effects models.
